# Limited Effects of High-Intensity Laser Therapy on Clinical and Radiographic Outcomes in Three-Year-Old Arabian Racehorses with Dorsal Metacarpal Disease

**DOI:** 10.3390/vetsci13070695

**Published:** 2026-07-17

**Authors:** Karolina Śniegucka, Maria Soroko-Dubrovina, Paulina Zielińska, Krzysztof D. Dudek

**Affiliations:** 1 Institute of Animal Breeding, Wroclaw University of Environmental and Life Sciences, 50-375 Wroclaw, Poland; maria.soroko@upwr.edu.pl; 2 Department of Surgery, Wroclaw University of Environmental and Life Sciences, 50-375 Wroclaw, Poland; paulina.zielinska@upwr.edu.pl; 3 Center for Statistical Analysis, Wroclaw Medical University, Ludwika Pasteura 1, 50-367 Wroclaw, Poland; krdudek@4wsk.pl

**Keywords:** dorsal metacarpal disease, HILT, Arabian racehorses

## Abstract

Dorsal metacarpal disease (DMD) is a common musculoskeletal injury in young racehorses, and effective non-invasive treatments remain of clinical interest. This study aimed to evaluate whether high-intensity laser therapy (HILT), which was applied as the sole intervention in horses retired from training, reduces the clinical signs of DMD compared with untreated controls. During the 2023–2024 racing seasons, 15 Arabian racehorses diagnosed with DMD were enrolled; 9 received HILT and 6 served as controls without laser therapy. The treatment protocol consisted of five daily HILT sessions followed by five sessions administered every other day. Thermographic, orthopedic, and radiographic examinations of the third metacarpal bones were performed before and after the treatment period in both groups, with additional thermographic and clinical assessments conducted throughout therapy. The results showed that horses treated with HILT did not exhibit a significant reduction in pain and lameness accompanied by thermographic changes consistent with decreased inflammation. These findings indicate that HILT did not alleviate clinical signs associated with DMD in the affected horses; however, further controlled studies are required to determine its effect on tissue healing processes and to optimize treatment duration.

## Simple Summary

Dorsal metacarpal disease is a common injury of the front limb bones in young racehorses that causes pain, inflammation and lameness, often reducing training capacity and performance. This study evaluated whether high-intensity laser therapy (HILT), a treatment that uses focused light energy to support tissue recovery, could reduce clinical signs of the disease compared with rest alone. Fifteen Arabian racehorses diagnosed with dorsal metacarpal disease were included in the study. All horses were withdrawn from training during the study period. Nine horses received a series of HILT sessions, whereas six horses served as untreated controls without additional therapy. All horses were regularly assessed using clinical examinations, thermographic imaging to measure surface temperature changes associated with inflammation, and radiographic evaluation of the affected bones. The results showed that horses treated with high-intensity laser therapy (HILT) did not demonstrate a significant reduction in pain and lameness, nor thermographic changes consistent with decreased inflammation. These findings indicate that HILT did not provide a measurable clinical benefit in alleviating signs associated with dorsal metacarpal disease in the affected horses under the conditions of this study. However, further controlled studies are required to clarify its potential effects on tissue healing processes and to optimize treatment duration.

## 1. Introduction

Dorsal metacarpal disease (DMD) is a well-recognized fatigue-related injury affecting young performance horses and is associated with decreased structural integrity of the dorsal cortex of the third metacarpal bone [1]. The condition is most commonly reported in racehorses during the early stages of training, with prevalence estimates reaching up to 70% in Thoroughbred horses entering intensive work [2]. Although historically linked primarily to Thoroughbreds, DMD has also been documented in purebred Arabian horses in clinical and radiological studies, indicating that the disorder is not breed-specific but rather reflects adaptive skeletal responses to repetitive mechanical loading and high-speed exercise [3]. It is attributed to cumulative mechanical stress leading to tissue failure [4,5], with repetitive biomechanical imbalances further exacerbating degenerative processes [6]. Lesions typically arise during periods of rapid increases in training intensity and are characterized by periosteal reactions affecting the dorsal and dorsomedial cortices of the third metacarpal bone, most often unilaterally [2,7,8,9]. Clinically, affected horses present with varying degrees of lameness, accompanied by localized heat, swelling, and marked pain on palpation of the dorsal surface of the third metacarpal bone [2,9].

The pathophysiology underlying these lesions closely parallels mechanisms described for stress fractures, which are considered multifactorial in origin. In cortical diaphyseal bone, remodelling processes associated with the resorption of accumulated microdamage are thought to transiently weaken bone tissue, thereby increasing susceptibility to stress fracture development [10]. Consistent with this mechanism, Katayama et al. [11] reported dorsomedial cortical fissures of the third metacarpal bone in Arabian horses subjected to high-intensity training protocols designed to induce DMD, accompanied by endocortical bone proliferation and underlying cortical radiolucency interpreted as evidence of active bone remodelling.

The standard management of horses affected by clinical DMD typically involves administration of phenylbutazone, application of ice therapy, and controlled hand-walking until the resolution of soreness, followed by a return to a modified training program [2]. Despite the widespread use of these approaches, the search for adjunctive therapies that may enhance tissue repair and shorten recovery time remains ongoing.

High-intensity laser therapy (HILT) has emerged as a promising non-invasive modality in equine veterinary medicine for the management of musculoskeletal and orthopedic conditions. The therapy has been shown to promote tissue regeneration, improve local blood flow, and stimulate cellular metabolic processes [12,13,14]. Studies in humans have demonstrated that HILT provides analgesic and anti-inflammatory effects, reduces pro-inflammatory mediators (PGE-2, TNF-α, IL-1β, IL-6), and exerts an anti-edematous effect through increased local blood flow and microvascular activation [15,16,17,18,19,20,21,22]. Additionally, HILT has been shown to stimulate fibroblast activity, enhance cellular metabolism, and promote tissue regeneration, including collagen synthesis and increased tendon tensile strength [14,16,23,24,25]. Santamato et al. [26] reported significant reductions in pain accompanied by improvements in muscle strength and functionality during HILT treatment.

Based on equine studies, HILT has demonstrated beneficial effects in the management of tendon and ligament injuries, resulting in reductions in pain, swelling, and lameness, as well as enhanced tissue healing [27,28,29]. Preliminary observations in cases of bone spavin also indicated decreased joint pain and improved lameness following HILT sessions [30]. Furthermore, our previous study on Thoroughbred horses with confirmed DMD demonstrated a reduction in pain response and lameness following HILT treatment [31].

Considering the documented effectiveness of HILT in promoting bone healing in human musculoskeletal disorders [32,33], this study was designed to evaluate its efficacy in the treatment of DMD, a stress-related condition characterized by periosteal inflammation and adaptive cortical bone remodeling in young racehorses. The study’s hypothesis was that HILT accelerates the healing process and reduces the duration based on thermographic examination, orthopedic examination and radiographic examination.

## 2. Materials and Methods

The study protocol was approved by the Local Ethics Committee for Animal Experiments in Wroclaw, Poland (No. 043/2023).

### 2.1. Animals and Data Collection

During the 2023 and 2024 racing seasons, 36 clinically sound three-year-old Arabian racehorses at the Partynice Racecourse in Wroclaw, Poland, were prospectively monitored for the development of DMD. In each season, the monitoring period extended from April to September. The inclusion of horses from two consecutive racing seasons was necessary because of the limited number of horses meeting the inclusion criteria within a single season. To ensure consistency between seasons and minimize management-related variability, all horses were trained by the same trainer according to an identical training program and were maintained under the same housing, feeding, and management conditions. All horses followed a standardized training program conducted six days per week, consisting of a 1000 m trot followed by 200–500 m of speed work. Owner consent was obtained from Partynice Racecourse (Wroclaw, Poland) before enrolment.

Before the monitoring period, all horses underwent comprehensive clinical and orthopedic examinations performed in accordance with established guidelines [34] to confirm soundness. The orthopedic evaluation included palpation of the third metacarpal bone and gait assessment on a hard surface. All examinations were performed by the same veterinarian with three years of clinical experience in equine orthopedics, and soundness was determined using an objective evaluation method [34].

The monitoring protocol included weekly thermographic examinations of the distal forelimbs, performed from the dorsal aspect to detect changes in skin surface temperature over the third metacarpal bone. At the beginning of the monitoring period, radiographic examinations of both forelimbs were performed in all horses. In addition, regular orthopedic examinations, including palpation for swelling, pain, and increased heat over the dorsal aspect of the third metacarpal bone, were carried out throughout the study by the same veterinarian.

Across both racing seasons, 15 of the 36 monitored horses developed clinical signs of DMD. Horses were eligible for inclusion in the treatment study if they were diagnosed with unilateral DMD based on the orthopedic examination and thermographic findings. Clinical signs included increased body surface temperature (temperature difference ≥ 1.25 °C, indicating subclinical inflammation [7]) and pain elicited on palpation of the dorsal surface of the third metacarpal bone. Horses presenting with wounds, traumatic lesions, disruption of skin continuity over the affected metacarpal bone, or other orthopedic conditions were excluded from the study.

The 15 eligible horses were randomly allocated to either the HILT treatment group (n = 9) or the untreated control group (n = 6) using a simple randomization procedure performed by the attending veterinarian. Throughout the treatment period, all horses were retired from training and maintained on strict box rest. None of the horses in either study group received pharmacological treatment or any other therapeutic intervention during the study. The only difference between the groups was that horses assigned to the HILT group underwent the HILT treatment protocol, whereas horses in the control group did not receive HILT.

The HILT protocol consisted of ten treatment sessions (five consecutive daily sessions followed by five sessions performed every other day), applied to the weight-bearing forelimbs while the horses were at rest. Thermographic and orthopedic examinations were performed immediately before and after each treatment session, and radiographic examinations were performed before the first and after the final treatment session. Horses in the control group underwent the same examination schedule without HILT treatment. The examiner responsible for outcome assessment remained blinded to group allocation.

For all clinical, thermographic, and radiographic assessments, measurements obtained from the DMD-affected forelimb were compared with those from the contralateral healthy forelimb within each study group.

### 2.2. Thermographic Examination

Thermographic assessments were performed using a calibrated VarioCam HR infrared camera (uncooled microbolometer focal plane array, 640 × 480 resolution, 7.5–14 μm spectral range, NETD < 20 mK at 30 °C, normal lens IFOV 0.57 mrad, ±1% measurement uncertainty; InfraTec, Dresden, Germany). Examinations followed a standardized protocol based on previous studies [22,35,36]. Ambient temperature during assessments ranged from 17 to 20 °C, measured with a TES 1314 thermometer (TES, Taipei, Taiwan).

Prior to imaging, horses underwent a 20-min acclimatization outside their stalls [37]. To minimize environmental interference, all assessments were conducted in an enclosed stable with windows closed. Any debris or artifacts on the dorsal forelimbs were removed by brushing one hour before imaging. All thermographic images were captured dorsally at a consistent camera-to-subject distance of 1.5 m by the same operator [38].

**Figure 1:**
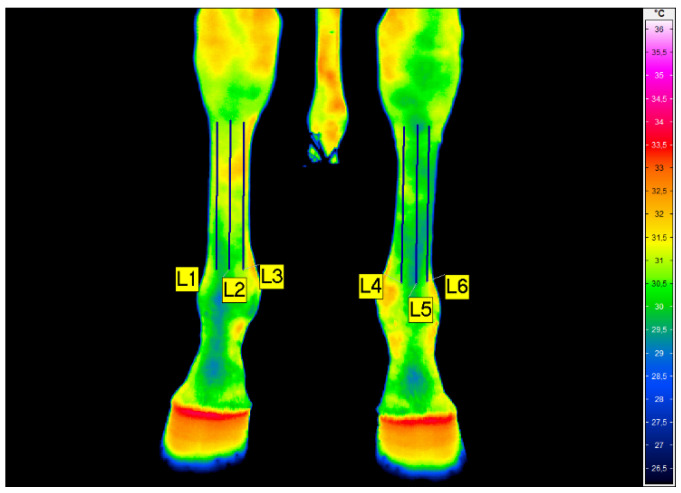
Representative thermographic images of the distal forelimbs (dorsal view) obtained before HILT in a horse from the HILT treatment group and a horse from the control group. The blue vertical lines represent the six linear regions of interest (line ROIs) used for temperature measurements. Three line ROIs were positioned on each third metacarpal bone, corresponding to the lateral, central, and medial regions. The paired ROIs were designated as L1/L6 (lateral), L2/L5 (central), and L3/L4 (medial). Temperature was measured separately within each of the six line ROIs (L1–L6).

Temperature analysis was performed manually by a single examiner using IRBIS 3 Professional software (InfraTec, Dresden, Germany). Measurements were taken from six linear regions of interest (ROIs) positioned on the dorsal surface of the third metacarpal bones of each forelimb: lateral (L1 and L6), central (L2 and L5), and medial (L3 and L4). Vertical reference lines ensured accuracy and minimized measurement error (Figure 1).

### 2.3. Orthopedic Examination

Orthopedic examinations were performed immediately after thermography and included palpation and lameness scoring. Palpation targeted the dorsal surface of the third metacarpal bones to detect swelling, heat, or pain. Both forelimbs were palpated in weight-bearing (proximal to distal) and non-weight-bearing (distal to proximal) positions [39]. Pain on palpation of the dorsal aspect of the third metacarpal bone was assessed using a modified 4-point ordinal scale (0–3), adapted from the composite orthopedic pain assessment described by Bussières et al. [12]: 0 = no reaction to palpation (no pain); 1 = mild avoidance response to palpation; 2 = resistance or withdrawal of the forelimb during palpation; 3 = marked withdrawal or pronounced avoidance response indicating severe pain.

Forelimbs showing a positive pain response were considered positive; those without response were classified as painless.

Lameness was evaluated using the 5-point scale of the American Association of Equine Practitioners [40,41]. Horses were assessed visually on a hard surface during straight-line walk and trot and during lunging in both directions. All assessments were performed by the same veterinarian to ensure consistency.

### 2.4. Radiographic Examination

Radiographic assessments were conducted using a digital system comprising a meX + 20 BT lite generator (Medical ECONECT, Oberhausen, Germany) and a DR detector PRIMOS 1210T MOD (Vieworks, Anyang-si, Republic of Korea). Lateromedial radiographs of the third metacarpal bones were obtained immediately after the orthopedic examination. Horses bore weight evenly on both forelimbs during imaging. The detector was positioned laterally between the forelimbs, with the generator directing X-rays perpendicularly onto the detector. The entire third metacarpal bone was included in the field of view.

Dorsal cortical thickness was measured at mid-length by the same examiner using VXvue software (Vieworks, Republic of Korea; https://xrayimaging.vieworks.com/en/software/vxvue, accessed on 12 July 2026) [7]. Changes in dorsal cortical thickness were recorded for each horse throughout the study.

### 2.5. High-Intensity Laser Therapy (HILT)

HILT was administered using a Class 4 laser system, Polaris HP S (ASTAR, Bielsko-Biała, Poland), which delivers two infrared wavelengths simultaneously: 808 nm (AlGaAs laser, 8 W) and 980 nm (InGaAs/AlGaAs laser, 10 W). Treatment was performed according to a standardized protocol based on previously published studies [7,35,42]. The energy density was set to 10.0 J/cm^2^ at a power output of 3.50 W, with a pulse frequency of 50 Hz and a total energy dose of 1000 J. Each treatment area measured 100 cm^2^, resulting in a treatment time of 5 min 57 s per forelimb, which was automatically calculated by the device.

The laser was applied using a continuous contact technique with the applicator held perpendicular to the treatment area and moved continuously to minimize light scattering and prevent local thermal effects. Therapy was performed with the horses standing square and bearing weight evenly on all four limbs. The dorsal surface of both third metacarpal bones was treated, and the coat was not clipped before treatment. The treatment protocol was identical for all horses.

All HILT sessions were performed by the same examiner, and the total treatment period for each horse was 15 days.

### 2.6. Statistical Analysis

All statistical analyses were performed using STATISTICA v. 13.3 (TIBCO Software Inc., Palo Alto, CA, USA), JASP (Version 0.19.3, JASP Team 2024), and Microsoft Excel. Prior to the statistical analyses, data distribution was assessed using the Shapiro–Wilk test, and homogeneity of variances was evaluated using Levene’s test. For descriptive purposes, continuous variables are presented as mean ± standard deviation (SD), median (interquartile range, IQR), and minimum–maximum range to provide a comprehensive description of the data distribution. Since the assumptions for parametric testing were not met, non-parametric statistical methods were applied. The Wilcoxon signed-rank test was used to compare cortical thickness between the DMD-affected forelimb and the contralateral healthy forelimb. To account for inter-individual variability and session-related fluctuations in ambient temperature, the analysis was performed using the difference in mean temperature between the DMD-affected and contralateral healthy forelimb for each of the 15 horses. The Wilcoxon signed-rank test was additionally used for these comparisons. The Mann–Whitney U test was applied to compare cortical thickness between the HILT treatment group and the control group. A *p*-value < 0.05 was considered statistically significant. Due to the ordinal nature of the data and repeated measurements within individual horses, a cumulative link model (CLM) was applied to assess tenderness of the dorsal metacarpal region while accounting for within-subject dependency.

## 3. Results

Horses in the HILT treatment group (N = 9) and control group (N = 6) differed significantly in cortical thickness of the forelimb affected by DMD at baseline (U = 9, *p* = 0.039). This difference remained and became more pronounced after therapy, with significantly higher values observed in the HILT treatment group compared to the control group at the post-treatment stage (U = 5, *p* = 0.011). No significant differences between groups were found for the healthy forelimb, either at baseline (*p* = 0.35) or after treatment (*p* = 0.22).

Within the HILT treatment group, no significant changes were detected in cortical thickness over time in either the DMD-affected forelimb (*p* = 0.59) or the sound forelimb (*p* = 0.51) (Table 1).

In the control group, cortical thickness parameters of both forelimbs remained unchanged over time. A decrease in values was observed in the DMD-affected forelimb (W = 1.5, *p* = 0.06), but this did not reach statistical significance (*p* = 0.05).

Cumulative link model (CLM) analysis (Table 2) showed a significant effect of time on forelimb tenderness, with improvement at day 15 (*p* = 0.0046). No significant group × time interaction was found (*p* > 0.05).

Threshold coefficients indicated separation between the “Non-painful” category and higher tenderness levels (Estimate = −3.23, z = −3.71), while the threshold between “Unchanged” and “Tender” was lower (Estimate = −0.07).

Time-related estimates were negative (e.g., −3.310), indicating a decrease in higher tenderness scores over time. Baseline tenderness was comparable between groups, and no significant differences in recovery patterns were observed (*p* = 0.66–1.00).

Descriptive statistics of body surface temperatures of the third metacarpal bones on the dorsal side, measured along lines L1 to L6 in horses from the HILT treatment group and control group on successive days of the experiment, are presented in Table 3 and Table 4.

Mean surface temperatures (Tavg) in symmetrical regions of interest (ROIs) of the DMD-affected forelimb (FD) and healthy forelimb (HF) at baseline are presented in Table 3. No significant differences between forelimbs were observed in any region (L1/L6, L2/L5, L3/L4).

Significant within-forelimb regional differences were found in both forelimbs (*p* < 0.001 for FD; *p* = 0.002 for HF), indicating a proximal-to-distal temperature gradient, with lower proximal (L1/L6: ~30.3 °C FD; 30.0 °C HF) and higher distal values (L3/L4: ~31.0 °C FD; 30.7 °C HF).

At session 10 (Table 4), a significant difference between forelimbs was detected in the proximal region (L1/L6), with higher temperatures in the FD (*p* < 0.05), while no differences were found in the middle or distal regions (*p* = 0.26 and *p* = 0.37).

At this stage, the gradient along the HF was no longer significant (*p* = 0.29), whereas the FD showed a borderline proximal-to-distal gradient (*p* = 0.05). Overall, the initial gradient diminished over time, with a localized proximal temperature increase observed in the affected forelimb.

## 4. Discussion

The present study evaluated whether HILT, applied as the sole therapeutic intervention in horses retired from training, accelerates clinical and structural recovery in Arabian racehorses with dorsal metacarpal disease. Overall, the results demonstrated that although forelimb tenderness decreased progressively during the study period and localized thermographic changes consistent with tissue remodeling were observed, HILT did not significantly accelerate clinical improvement or periosteal remodeling compared with untreated horses. Therefore, the study hypothesis that HILT would accelerate the healing process and shorten recovery time was not confirmed under the conditions of the present study [43].

Analysis of cortex thickness indicated notable differences between the HILT and non-HILT groups at baseline, reflecting inherent variability in the severity or stage of DMD-related changes. This initial heterogeneity should be considered when interpreting post-treatment outcomes, as it may partially explain the persistence of higher values in the HILT group following therapy. Increased periosteal thickness is generally recognized as a hallmark of adaptive bone response and active remodeling rather than a direct sign of acute inflammation [44,45].

During the observation period, no significant pre- to post-treatment changes in periosteal thickness were detected within the HILT group, suggesting that high-intensity laser therapy did not induce rapid structural changes in the periosteum. The HILT protocol applied in the present study was evaluated in the context of previously published HILT studies; however, differences in treatment parameters, target tissues, and outcome measures limit direct comparison between studies [17,29,33]. Nevertheless, the present findings are consistent with the proposed mechanism of photobiomodulation, which primarily enhances local microcirculation, modulates inflammation, and stimulates cellular metabolism, thereby promoting tissue repair rather than producing immediate structural changes detectable by radiography [29]. Similar observations have been reported in horses treated with HILT for tendon and ligament injuries, where the therapy promoted tissue healing and clinical recovery without immediate morphological remodeling of the affected tissues [29]. Likewise, studies in patients with knee osteoarthritis demonstrated that HILT effectively reduced pain and improved joint function, whereas structural tissue changes were not expected over the short observation period [17,33]. Therefore, the absence of a significant reduction in periosteal thickness in the present study should not be interpreted as evidence of a lack of biological effect. Instead, it is likely that HILT facilitated biological processes involved in tissue repair, while measurable structural remodeling of the periosteum requires a longer period to become radiographically apparent. Furthermore, the tendency toward decreased periosteal thickness observed in the non-HILT group may reflect the natural resolution of overload-induced periosteal reactions following cessation of training, consistent with the physiological process of bone remodeling under reduced mechanical loading [46].

The absence of differences in the healthy forelimb supports the localized character of these changes and indicates that the measured effects were confined to the affected region rather than reflecting systemic influences. Collectively, these findings suggest that periosteal thickness responds gradually to therapeutic interventions and primarily reflects ongoing remodeling activity, rather than serving as a short-term marker of treatment efficacy. This emphasizes the value of combining structural and functional assessments when evaluating interventions such as HILT in horses with DMD.

Forelimb tenderness in horses with DMD decreased progressively over time, with clinical improvement becoming evident from day 15 onward. The absence of a significant group × time interaction suggests that both HILT-treated and non-treated horses exhibited comparable trajectories of pain reduction. This pattern aligns with previous studies indicating that soft tissue discomfort associated with overload injuries tends to ameliorate progressively as part of the natural healing response and musculoskeletal adaptation to training [34,47]. The similar temporal profile observed across groups may therefore reflect inherent biological repair mechanisms rather than a distinct analgesic effect attributable to laser therapy. However, our previous study demonstrated a reduction in pain and lameness in horses treated with HILT. These findings suggest that HILT may alleviate clinical signs associated with DMD [31].

The time-dependent reduction in the probability of higher tenderness scores indicates a gradual decrease in forelimb discomfort over the follow-up period. Improvement was evident from day 15 in both groups, suggesting a consistent recovery pattern over time. The clear separation between the “non-painful” category and higher tenderness levels indicates that this change was clinically meaningful rather than reflecting random score variation, in agreement with validated equine pain assessment approaches [48]. However, the absence of a significant group × time interaction, together with comparable recovery trajectories between groups, indicates that high-intensity laser therapy did not modify the overall pattern of clinical improvement compared with rest alone. The smaller differentiation between intermediate tenderness categories further highlights the limited sensitivity in detecting subtle clinical changes during early recovery, a limitation previously reported in musculoskeletal rehabilitation studies, where early functional improvements may be present despite minimal observable clinical differences [49].

Taken together, these results indicate that although forelimb tenderness significantly decreased over the study period, high-intensity laser therapy did not demonstrate a measurable effect on the rate of pain resolution in horses with DMD under the conditions evaluated. The observed reduction in pain appears to be consistent with the expected course of tissue adaptation and healing associated with controlled exercise and rest. These findings suggest that natural recovery processes may play a primary role in the resolution of exercise-induced musculoskeletal discomfort in equine athletes within the context of this study.

Thermographic assessment in the present study revealed temporal changes in temperature distribution that extend beyond simple comparisons of asymmetry between affected and healthy limbs. The absence of significant baseline differences suggests that early DMD-associated changes may not manifest as marked surface thermal asymmetry, either because acute inflammation was not present at the time of initial evaluation or because physiological thermoregulatory mechanisms compensated for subtle thermal deviations. Such subclinical inflammatory states may not be readily detectable with surface thermography alone, as previously described in equine exercise-induced overload where latent tissue responses do not immediately alter surface heat patterns [44,50]. The observed proximal-to-distal gradient in both limbs at baseline is consistent with normal thermal distribution patterns related to vascular supply and anatomical conformation [50].

In later sessions, a localized increase in temperature was observed on the dorsal surface of the metacarpal bone. This change may indicate a focal metabolic response associated with tissue remodeling processes rather than a generalized inflammatory reaction. The elevated surface temperature likely reflects increased local perfusion and metabolic activity underlying bone adaptation, which is consistent with reports indicating that thermography can detect localized tissue responses during active remodeling phases [29]. Concurrent normalization of thermal patterns in the contralateral healthy limb may reflect gradual adaptation to consistent mechanical loading, whereas the persistence of regional thermal differences in the affected limb highlights ongoing local tissue activity.

These observations highlight the usefulness of thermography as a non-invasive tool for monitoring the spatial redistribution of tissue activity during the remodeling phase of DMD, as well as a potential indicator of subclinical inflammatory changes. By capturing subtle temporal variations in thermal patterns, thermography may serve as a valuable adjunct to clinical and imaging modalities in assessing tissue responses to mechanical loading and adaptive processes.

Several limitations should be acknowledged. The relatively small sample size may have limited the statistical power to detect subtle treatment effects, particularly in longitudinal analyses. Baseline differences in cortical thickness, surface temperature, and pain between the HILT treatment group and the control group introduced partial heterogeneity, which may have influenced post-treatment comparisons. Furthermore, radiographic assessment was limited to latero-medial projections. Although additional oblique projections could have improved the visualization of cortical bone changes and periosteal reactions associated with dorsal metacarpal disease, they were not included because consistent reproduction of identical oblique positioning during serial examinations would have been difficult, potentially reducing the reliability of longitudinal cortical thickness measurements. Outcome measures represented distinct biological processes—functional (pain), physiological (thermography), and structural (cortical thickness)—which evolve at different temporal scales, limiting the ability of short-term observation to fully capture tissue remodeling. Thermographic measurements are additionally influenced by physiological thermoregulation and environmental factors, potentially masking subtle changes. Finally, the absence of long-term follow-up precludes assessment of whether the observed improvements translated into sustained functional or orthopedic benefits.

Future studies with larger cohorts, randomized allocation, and extended follow-up combining clinical, imaging, and performance outcomes are warranted to clarify the long-term role of HILT in DMD management.

This study demonstrated that horses with DMD in both groups exhibited a progressive reduction in forelimb tenderness over the course of the study, highlighting the contribution of natural healing and adaptive processes rather than an acceleration of recovery attributable to HILT. Thermography revealed a localized increase in temperature on the dorsal surface of the metacarpal bone, which may be consistent with visualization of an acute inflammatory process. Periosteal thickness measurements indicated gradual structural adaptation, whereas high-intensity laser therapy did not significantly accelerate either clinical or structural recovery, although it may modulate local biological activity. These findings suggest that the healing process in DMD results from an interaction between natural adaptation and local tissue responses, and that thermography and periosteal assessment are useful tools for monitoring both inflammatory processes and bone remodeling.

## 5. Conclusions

Horses with DMD exhibited a progressive reduction in forelimb tenderness over time, indicating that natural healing and adaptive processes are key contributors to recovery. High-intensity laser therapy did not significantly accelerate clinical or structural improvement under the conditions of this study. Changes in periosteal thickness reflected ongoing bone remodeling, while thermography demonstrated dynamic redistribution of tissue activity consistent with adaptive responses and possible subclinical inflammatory changes. Overall, recovery in DMD appears to be primarily driven by intrinsic repair mechanisms associated with controlled exercise and rest. The combined use of clinical, thermographic, and structural assessments provides a useful approach for monitoring disease progression and tissue adaptation in equine athletes. Further long-term studies are required to better define the role of high-intensity laser therapy in the management of DMD in Arabian Racehorses.

## Figures and Tables

**Table 1:** Comparison of forelimb cortical thickness parameters in the HILT treatment group (N = 9) and the control group (N = 6) before and after treatment. Data are presented as median (interquartile range).

Session	Forelimb	Groups		Mann–Whitney U Test
		HILT Treatment Group *N* = 9	Control Group *N* = 6	
Baseline	With DMD	13.0 ± 1.2 12.8 [12.3, 13.4] 10.9–15.2	11.8 ± 0.8 11.7 [11.3, 12.3] 10.6–12.8	*U* = 9 ***p***** = 0.039**
	Healthy	13.2 ± 1.7 13.7 [11.5, 14.5] 11.0–15.3	12.1 ± 0.7 12.0 [11.5, 12.6] 11.3–13.2	*U* = 18.5 *p* = 0.35
	Wilcoxon signed-rank test	*W* = 18 *p* = 0.59	*W* = 6 *p* = 0.35	×
Post-treatment	with DMD	13.3 ± 1.0 13.3 [12.3, 14.1] 11.9–15.0	11.6 ± 0.8 11.4 [10.9, 12.3] 10.7–12.8	*U* = 5 ***p***** = 0.011**
	Healthy	13.0 ± 1.5 13.6 [11.9, 13.9] 10.6–15.2	12.0 ± 0.6 12.0 [11.6, 12.6] 11.1–12.8	*U* = 16 *p* = 0.22

**Table 2:** Results of the cumulative link model (CLM) analysis for forelimb tenderness scores in horses, with fixed effects of treatment group (HILT treatment group vs. control group), time (categorical), and their interaction (Pain_Score ~ Group × Time).

Coefficients	Estimate	Std. Error	*z* Value	Pr (>|*z*|)
HILT treatment group	0.203	1.026	0.198	0.843
Day 2	0.000	1.116	0.000	1.000
Day 3	0.000	1.116	0.000	1.000
Day 4	0.000	1.116	0.000	1.000
Day 5	−1.025	1.167	−0.878	0.380
Day 15	−3.310	1.167	−2.836	**0.005**
HILT treatment group × Day 2	−0.000	1.450	0.000	1.000
HILT treatment group × Day 3	0.000	1.450	0.000	1.000
HILT treatment group × Day 4	−0.640	1.459	−0.438	0.661
HILT treatment group × Day 5	−0.391	1.520	−0.257	0.797
HILT treatment group × Day 15	0.233	1.459	0.159	0.873
Threshold coefficients vs				
Non-painful|Unchanged	−3.234	0.871	−3.713	
Unchanged|Tender	−0.076	0.789	−0.096	

**Table 3:** Mean surface temperature (Tavg, °C) measured in symmetrical linear regions of interest (line ROIs) located on the dorsal surface of the third metacarpal bone in the DMD-affected forelimb (FD) and the contralateral healthy forelimb (HF) of horses from the HILT treatment group at baseline (Session I). Data are presented as mean ± SD, median [IQR], and minimum–maximum range.

Session	ROIs	HILT Treatment Group N = 9		Wilcoxon Signed-Rank Test
		FD	HF	
I	L1/L6	30.3 + 1.3	30.0 + 1.4	*W* = 10 *p* = 0.14
		30.2 [29.5; 31.4]	29.9 [29.3; 31.1]	
		28.1–31.8	27.4–31.7	
	L2/L5	30.6 + 1.2	30.4 + 1.4	*W* = 10 *p* = 0.26
		30.3 [29.7; 31.6]	30.5 [29.7; 31.6]	
		28.6–32.5	27.7–32.1	
	L3/L4	31.0 + 1.2	30.7 + 1.3	*W* = 11 *p* = 0.33
		31.1 [30.1; 31.9]	31.0 [30.0; 31.6]	
		29.1–32.8	28.2–32.8	
Friedman test		χ^2^ = 16.2 ***p***** < 0.001**	χ^2^ = 12.7 ***p***** = 0.002**	×

**Table 4:** Mean surface temperature (Tavg, °C) measured in symmetrical linear regions of interest (ROIs) located on the dorsal surface of the third metacarpal bone in the DMD-affected forelimb (FD) and the contralateral healthy forelimb (HF) of horses in the HILT treatment group. Data are presented as mean ± SD, median (IQR), and minimum–maximum range. X denotes the session number.

Session (Time)	ROIs	HILT Treatment Group N = 9		Wilcoxon Signed-Rank Test
		FD	HF	
X	L1/L6	29.6 ± 3.7	29.2 ± 3.5	*W* = 5 ***p***** = 0.038**
		30.0 [29.6; 32.9]	29.7 [27.9; 32.6]	
		23.6–33.6	23.8–32.9	
	L2/L5	30.0 ± 3.2	29.7 ± 2.8	*W* = 13 *p* = 0.26
		30.2 [29.3; 32.9]	30.1 [27.7; 32.6]	
		24.2–33.8	25.2–32.9	
	L3/L4	30.3 ± 3.3	29.8 ± 3.0	*W* = 15 *p* = 0.37
		30.4 [29.4; 33.4]	30.5 [27.9; 32.6]	
		24.6–34.1	24.5–32.9	
Friedman test		χ^2^ = 6.0 ***p***** = 0.05**	χ^2^ = 2.5 *p* = 0.29	×

## Data Availability

The original contributions presented in this study are included in the article. Further inquiries can be directed to the corresponding author.
